# Role of a Novel Silver Fir (*Abies alba*) Extract, Abigenol^®^/AlbiPhenol^®^, in Modulating Cardiovascular Disorders: Key Factors

**DOI:** 10.3390/antiox11040618

**Published:** 2022-03-23

**Authors:** Kevin Leone, Marta Micheletto, Giovanni Di Maira, Erik Tedesco, Federico Benetti, Urška Zaloker

**Affiliations:** 1 ECSIN-European Center for the Sustainable Impact of Nanotechnology, ECAMRICERT SRL, 35127 Padova, Italy; k.leone@ecamricert.com (K.L.); m.micheletto@ecamricert.com (M.M.); g.dimaira@ecamricert.com (G.D.M.); e.tedesco@ecamricert.com (E.T.); f.benetti@ecamricert.com (F.B.); 2 Ars Pharmae, Ltd., Litostrojska Cesta 46A, SI-1000 Ljubljana, Slovenia

**Keywords:** *Abies alba*, silver fir bark extract, cardioprotective, oxidative stress

## Abstract

Cardiovascular diseases (CVDs) represent the leading cause of death worldwide, being responsible for about one third of deaths. Among CVDs, coronary artery diseases (CADs) are characterized by vascular endothelium dysfunction due to oxidative and inflammatory damages, the oxidation of circulating low-density lipoproteins (LDL) and high-density lipoproteins (HDL), and the production of ROS in the steatotic liver with the consequent increase of lipids and cholesterol. Together with CADs, heart failure (HF) represents another high-mortality rate CVD. A major risk factor for HF is hypertension that is accompanied by oxidative stress. Phytoextracts, rich in antioxidant and anti-inflammatory compounds, may have therapeutic value as they can interfere with several CVDs risk factors. In this work, a novel silver fir (*Abies alba*) bark extract, Abigenol^®^/AlbiPhenol^®^, was studied. First, Abigenol^®^/AlbiPhenol^®^ cytotoxicity, bioaccessibility and bioavailability were evaluated by using an in vitro digestion model. Abigenol^®^/AlbiPhenol^®^ was shown to be non-cytotoxic and showed good bioaccessibility. Then, by using in vitro hepatic, cardiac and vascular models, its antioxidant and anti-steatotic properties were assessed. Abigenol^®^/AlbiPhenol^®^ showed an effective antioxidant action, and it was able to inhibit LDL and HDL oxidation, the main actors in atherosclerotic plaque formation. In steatotic conditions, Abigenol^®^/AlbiPhenol^®^ induces decreased lipid and cholesterol accumulation in hepatocytes. In addition, in a cardiac model, the formulation reduced the activity of the hypertension-related angiotensin-converting enzyme (ACE). Altogether, these findings reveal a potential application of Abigenol^®^/AlbiPhenol^®^ in the prevention and treatment of CVDs.

## 1. Introduction

Cardiovascular diseases (CVDs) are a class of disorders that involve the blood vessels and heart. In recent years, CVDs have become the leading causes of morbidity and mortality worldwide. According to data provided by the World Health Organization (WHO), CVDs account for 31% of annual global deaths [1]. Among CVDs, coronary artery diseases (CADs) are characterized by atherosclerosis that consists of a progressive build-up of plaque on the inner artery walls, leading to artery lumen narrowing and blood flow slowdown [2,3]. Despite its complex etiology, it is generally agreed that endothelial dysfunction is involved in the onset and progression of atherosclerotic CVDs [4,5,6]. Indeed, the vascular endothelium, a single layer of cells lining the lumen of blood vessels, is endowed with many relevant physiological functions, such as the regulation of blood vessel diameter, blood pressure, inflammatory state and water permeability [7,8].

Inflammation is an innate immune system reaction that aims to maintain a homeostatic internal milieu while being exposed to environmental stresses. The inflammatory response is focused on reducing agents leading to injury and/or correlated effects, with the aim to restore the homeostasis of damaged tissues. The initiation of inflammation responses involves innate sensing mechanisms that detect the bacterial infection, stressed or dying cells, cellular integrity loss, barrier leakage, etc. The eradication of inflammatory causes occurs through a cascade of inflammatory pathways and mechanistic effects well-orchestrated by the immune system. Different types of immune cells are able to change their number, morphology, and phenotype depending on the type and stage of inflammation. From the molecular point of view, the inflammation process is characterized by a local increase of tissue hormones, components of the complement, cytokines, and lipid mediators. The majority of these products are synthesized at the site of inflammation and contribute to resolving the inflammatory process by removing or inhibiting the biological activity of the triggering agent. If causative agents persist, a chronic inflammatory process induces further tissue dysfunction and detrimental consequences [9]. Several risk factors for chronic diseases such as hypertension, diabetes, smoking, excessive food intake, underlying autoimmune diseases, pollution and genetic abnormalities [10] are due to chronic and unresolved inflammatory manifestations. As such, chronic oxidative and inflammatory damage could lead to endothelial dysfunction, potentially causing the formation of atherosclerotic plaques [11,12]. Together with vascular endothelium injury, atherosclerosis can also be caused by the local overexpression of inflammatory mediators, oxidation of circulating low-density lipoproteins (LDL) and high-density lipoproteins (HDL) or high levels of lipids and cholesterol circulating in the bloodstream [13]. The latter alteration can be the consequence of liver dysfunctions such as steatosis, represented by the progressive accumulation of fats and lipids. For example, in non-alcoholic fatty liver disease (NAFLD), fat and lipids accumulate in the liver due to a sedentary lifestyle, high-fat diet, obesity and insulin resistance in genetically predisposed individuals, and progressively lead to steatosis, non-alcoholic steatohepatitis and cirrhosis. Steatosis consists of hepatic de novo lipogenesis that, in combination with the reduction of lipolysis adipose tissue lipolysis, induces an increase of liver fatty acids [14,15]. Adipose tissue dysfunction and fat accumulation in the liver, especially triglycerides, also cause oxidative stress due to reactive oxygen species (ROS) production, as well as endoplasmic reticulum and mitochondrial dysfunction [16]. In pathological conditions, the liver is not able to properly regulate lipids and cholesterol homeostasis, with a subsequent increase in their circulating levels. This effect represents one of the early events in the formation of atherosclerotic plaques, effectively linking an altered lipid homeostasis in the liver to CADs [13,16,17]. In addition to CADs, heart failure (HF) is a CVD involving more than 26 million people worldwide, with an increasing tendency [18,19]. From a medical point of view, HF is a complex clinical syndrome, characterized by an insufficient cardiac output or by an adequate cardiac output secondary to compensatory chronic activation of the sympathetic nervous system and the renin–angiotensin–aldosterone system (RAAS). The latter, known as neurohormonal compensation, is the major mechanism underlying the progression of HF [20,21]. As for the other CVDs, HF etiology is complex due to the numerous factors involved in its onset and development. However, it is commonly agreed that hypertension, defined as elevated blood pressure, is a major risk factor for HF [22,23]. In physiological conditions, blood pressure is strictly regulated, in a short-term fashion, by baroreceptors located in specific key points (i.e., high-pressure and low-pressure receptor zones), while long-term regulation is mainly carried out via the renin–angiotensin system (RAS) [24]. It is not surprising that hypotensive drugs, like lisinopril, captopril or enalapril, target key RAS processes, such as the conversion of angiotensin I to vasoactive angiotensin II by the angiotensin-converting enzyme (ACE) [25]. The scientific literature from recent years has identified oxidative stress as a pivotal player in the onset of CVDs and, in particular, of CADs and HF. In the case of CADs, three mechanisms linked to oxidative stress have already been cited above as potential causes for atherosclerosis: the oxidative damages to the vascular endothelium, the oxidation of circulating LDL/HDL, and the steatosis-induced production of ROS in the liver with a consequent increase in the circulating levels of lipids and cholesterol. Oxidative stress has also been related to hypertension pathogenesis and, as a consequence, to HF [26,27]. Indeed, hypertension is accompanied by decreased levels of ROS- and reactive nitrogen species (RNS)-scavenging systems (i.e., catalase and/or superoxide dismutase and glutathione), decreased nitric oxide (NO) production and increased levels of the pro-oxidant hydrogen peroxide [28,29]. Interestingly, a correlation between elevated oxidative stress and renin activation leading to hypertension was highlighted [30]. Consequently, considering the tight connection between oxidative stress and steatosis with atherosclerosis and hypertension, compounds characterized by strong antioxidant, anti-steatotic and anti-inflammatory properties could be instrumental in preventing and treating CVDs like CADs and HF [31]. Thanks to their composition, rich in antioxidant and anti-inflammatory compounds (phenols, polyphenols, catechins, epicatechins, quercetin, etc.), bark extracts have catalyzed the attention of many pharmaceutical, food supplement and cosmetic industries [32,33]. Recently, a novel bark extract from silver fir (*Abies alba*), with an already-proven antioxidant effect [34], has been marketed with the name Abigenol^®^/AlbiPhenol^®^. In the present study, in order to assess the potential health benefits of Abigenol^®^/AlbiPhenol^®^ on CVD prevention and treatment, its bioaccessibility and bioavailability was evaluated, and then, by using in vitro hepatic, cardiac and vascular models, the antioxidant, anti-steatotic and anti-hypertensive properties were investigated. 

## 2. Materials and Methods

### 2.1. Materials

Caco-2 human colon adenocarcinoma cells, HepG2 human hepatocytes, H9c2 myoblasts from rat heart and HUVEC human umbilical vein endothelial cells were all purchased from ATCC (Manassas, VA, USA, respectively cat. n. HTB-37, HB-8065, CRL-1446 and PCS-100-010). DMEM + GlutaMAX (Dulbecco’s modified Eagle’s medium) was purchased from GIBCO, while fetal bovine serum (FBS), penicillin/streptomycin solution and L-glutamine were purchased from Lonza (Basel, Switzerland). Medium 200, Low Serum Growth Supplement (LSGS), human low-density lipoprotein (LDL) and a Micro BCA Protein Assay Kit were purchased from Thermo Fisher Scientific (Waltham, MA, USA). Hank’s balanced salt solution (HBSS), high-glucose Dulbecco’s Modified Eagle’s Medium (DMEM), non-essential amino acids (NEAA), bovine serum albumin (BSA), Lucifer Yellow (LY), human high-density lipoprotein (HDL), 4′,6-diamidino-2-phenylindole (DAPI), Nile Red, oleic acid, diethylmaleate (DEM), Millicell hanging cell culture inserts, Lisinopril, CelLytic M, 2′,7′-dichlorofluorescin diacetate (DCFDA), a superoxide dismutase (SOD) activity assay kit and an angiotensin-converting enzyme (ACE) activity assay kit, a Cholesterol Quantitation Kit, a Bile Acid Assay Kit and a Triglyceride Quantification Kit were purchased from Merck KGaA (Darmstadt, Germany). CellTiter 96^®^ AQueous One Solution Cell Proliferation Assay (MTS) and GSH/GSSG-Glo™ Assay were purchased from Promega (Madison, WI, USA), Bradford protein assay from Bio-Rad (Hercules, CA, USA), Oxygen Radical Antioxidant Capacity (ORAC) Assay from Cell Biolabs (San Diego, CA, USA) and C18 chromatographic columns from Agilent (Santa Clara, CA, USA).

### 2.2. Cell Cultures

#### 2.2.1. Caco-2 Cell Culture

Caco-2 cells (passage 30 to 40) were maintained in cell culture medium (Caco-2 CCM) (DMEM High Glucose medium supplemented with 10% FBS, 2% L-glutamine, 1% NEAA, and 1% penicillin–streptomycin mix). The cells were grown in a cell culture incubator (85% relative humidity, 5% CO_2_ and 37 °C). The Caco-2 cells were seeded at 2000 cell/cm^2^ and the medium changed every other day. The cells were subcultivated by treatment with trypsin every 7 days when they reached 80–90% confluency.

#### 2.2.2. HepG2 Cell Culture

HepG2 cells (passage 90 to 95) were maintained in cell culture medium (HepG2 CCM) (DMEM + GlutaMAX medium supplemented with 10% FBS and 1% penicillin–streptomycin mix). The cells were grown in a cell culture incubator (85% relative humidity, 5% CO_2_ and 37 °C). HepG2 cells were seeded at 15,000 cell/cm^2^ and the medium changed every other day. The cells were subcultivated by trypsinization every 4 days, when they reached 80–90% confluency.

#### 2.2.3. H9c2 Cell Culture

The rat myoblasts H9c2 (passages from 3 to 7) were maintained in H9c2 cell medium (H9c2 CCM) (high glucose DMEM supplemented with 10% FBS and, 100 U/mL penicillin/streptomycin, 2 mM L-glutamine, 1 mM sodium pyruvate 1% penicillin–streptomycin mix). The cells were grown in a cell culture incubator (85% relative humidity, 5% CO_2_ and 37 °C). H9c2 cells were seeded at 5000 cell/cm^2^ and the medium was changed every day. The cells were subcultivated by trypsinization when they reached 80–90% of confluency.

#### 2.2.4. HUVEC Cell Culture

The HUVEC human umbilical vein endothelial cells (passages from 7 to 16) were maintained in Medium 200 with a Low Serum Growth Supplement (LSGS) kit. The cells were grown in a cell culture incubator (85% relative humidity, 5% CO_2_ and 37 °C). HUVEC cells were seeded at 8000 cell/cm^2^ and the medium was changed every day. The cells were subcultivated by trypsinization when they reached 80–90% of confluency. 

### 2.3. Determination of Catechin Content within Abigenol^®^/AlbiPhenol^®^

Abigenol^®^/AlbiPhenol^®^ was analyzed for catechin content, intended as epigallocatechin gallate (EGCG) concentration. Reversed phase HPLC with UV detection (at 280 nm) was used to analyze EGCG. The HPLC separation was carried out at 25 °C using a Nexera XR HPLC system (Shimadzu) connected to a diode-array detector (DAD, Shimadzu). A C8 column (4.6 × 150 mm) was used with orthophosphate 0.1% in acetonitrile as an isocratic eluent. Each sample was dissolved in methanol using an ultrasonication bath and diluted in the same solvent. Quantification of the eluted EGCG was accomplished by the method of peak area using the range of calibration of 3 to 650 μg/mL of EGCG (Merck KGaA) as an external standard.

### 2.4. Determination of Abigenol^®^/AlbiPhenol^®^ Bioaccessibility

A single dose of Abigenol^®^/AlbiPhenol^®^ (150 mg) was exposed to an in vitro digestion simulating the physiological digestion in the oral, gastric and intestinal compartments. Briefly, the formulation was incubated for 5 min in saliva at 37 ± 1 °C and with a head-over-heels rotative movement at 55 rpm, simulating peristalsis. Subsequently, gastric juice (pH 1.3 ± 0.1) was added to the mixture; the pH of the sample was checked and, if necessary, adjusted to 2.5 ± 0.5 with NaOH (1 M) or HCl (37%). The sample was further incubated at 37 °C for 2 h. Subsequently, duodenal juice (pH 8.1 ± 0.1), bile (pH 8.2 ± 0.1) and sodium bicarbonate were added, and the pH of the mixture was set at 6.5 ± 0.5; the sample was rotated head-over-heels for other 2 h. The simulated digestive fluids composition refers to Walczak et al. [35]. Once the digestion process was complete, the bioaccessibility of Abigenol^®^/AlbiPhenol^®^ was determined by measuring the catechin epigallocatechin gallate (EGCG) by means of high-pressure liquid chromatography (HPLC).

### 2.5. In Vitro Model of Human Intestinal Epithelium

The absorption and bioavailability of Abigenol^®^/AlbiPhenol^®^ were determined through an in vitro model of human intestinal epithelium based on Caco-2 cells. Briefly, the Caco-2 cells were seeded on Millicell PTFE inserts (1 μm pore size) at an initial density of 150,000 cells/cm^2^ and allowed to mature and differentiate for 21 days. Indeed, thanks to the Millicell system design with the apical/luminal and basolateral/serosal compartments, Caco-2 cells differentiate and acquire morphological and functional features typical of enterocytes, such as the presence of microvilli, tight junctions and P-glycoproteins. Absorption experiments were performed between 21 and 28 days after seeding.

### 2.6. Evaluation of Abigenol^®^/AlbiPhenol^®^ Bioavailability

Based on the dose–response curve and barrier integrity, digested Abigenol^®^/AlbiPhenol^®^ was added to the apical side of the in vitro intestinal epithelium, while HBSS supplemented with 1% BSA was placed in the basolateral compartment. Due to the lipophilicity of the formulation’s main active components, the BSA was added to the basolateral compartment to improve the bioavailability. According to the literature [36], the addition of BSA improves the correlation between the absorption occurring in Caco-2 cell monolayers and in humans. After incubation of 1 and 3 h, solutions of apical and basolateral compartments were collected and their EGCG content was determined by HPLC. The bioavailability of Abigenol^®^/AlbiPhenol^®^, expressed as catechins, was indicated as a percentage of absorption after three independent experiments.

### 2.7. Evaluation of the Impact of Digested Formulations on the Intestinal Epithelium Viability

To evaluate the impact of digested Abigenol^®^/AlbiPhenol^®^ on intestinal epithelium viability, digested Abigenol^®^/AlbiPhenol^®^ was diluted serially in digestive fluids (from 1:2 up to 1:50 dilution) and added to the apical compartment of the in vitro intestinal epithelia, while HBSS buffer was placed in the basolateral compartment. Digestive fluids (without formulations) were added to the apical compartment of the in vitro intestinal epithelia as a negative control. After 3 h of incubation, the monolayers were washed twice with pre-warmed HBSS and the viability of the intestinal epithelia were evaluated with the MTS assay, according to the manufacturer’s instructions. This assay is based on the MTS tetrazolium reduction by viable cells to generate a colored formazan product that can be quantified by measuring the absorbance at 490 nm. The color intensity at 490 nm was measured with a microplate reader (Synergy4, Biotek, Winooski, VT, USA). Cell viability (%) was expressed as the ratio of the color intensity in the treated group on that in the control group (untreated). In parallel, the barrier integrity of the treated epithelia was determined by measuring the trans-epithelial electrical resistance (TEER), before and after the treatment and after 24 h of recovery, with an ERS2 Voltohmmeter (Millipore, Burlington, MA, USA). The bioavailability experiments were performed using non-toxic concentrations, determined by dose–response curves, which did not alter the barrier integrity of the epithelia.

### 2.8. Barrier Integrity and Cell Viability

After exposure to the digested formulations, the viability and barrier integrity of the intestinal epithelium model were evaluated. At the end of the incubation with digested Abigenol^®^/AlbiPhenol^®^, the epithelia were washed twice with pre-warmed HBSS and equilibrated with the same buffer for 30 min. Once equilibrated, their barrier integrity was evaluated by measuring the trans-epithelial electrical resistance (TEER) of the monolayer. The paracellular permeability of the model was determined with a Lucifer Yellow (LY) probe unable to pass through intact tight junctions. The paracellular permeability was measured by adding 0.5 mL of 100 μg/mL LY in HBSS in the apical compartment and 1.5 mL of HBSS in the basolateral compartment. After an hour, the basolateral fractions were collected and fluorescence measured with a spectrofluorometer (Synergy 4, Biotek).

The apparent permeability coefficient (Papp, cm/s) was calculated with the following formula:

*Papp* = (Δ*C* · *V*)/(Δ*t* · *A* · *C*0)

where ΔC/Δt is the flow of molecules being transported across the monolayer during the incubation time (mM/s), V is the volume of the basolateral compartment (cm^3^), A is the area of the membrane (cm^2^) and C0 is the initial concentration of the molecule in the apical compartment. Finally, cell viability was evaluated by using MTS assay according to the manufacturer’s instructions.

### 2.9. Evaluation of Abigenol^®^/AlbiPhenol^®^ Cytotoxicity

To evaluate the formulation impact on the viability of the in vitro models, the three cell lines were exposed to increasing concentrations of Abigenol^®^/AlbiPhenol^®^. CCM (without the formulation) was used as a negative control. After 24 h of incubation, cells were washed twice with pre-warmed HBSS and viability was evaluated by MTS assay, according to the manufacturer’s instructions. The absorbance at 490 nm was determined with a microplate reader (Synergy4, Biotek). Cell viability (%) was expressed as the ratio of the color intensity in the treated groups to that in the control (untreated) group. Efficacy experiments were subsequently performed considering the bioavailable and the highest non-toxic concentrations of Abigenol^®^/AlbiPhenol^®^.

### 2.10. Determination of Antioxidant Activity of Abigenol^®^/AlbiPhenol^®^ on Cultured Cells

The protective impact of Abigenol^®^/AlbiPhenol^®^ on the oxidative state of the three cell lines was assessed by means of three different assays. Briefly, following seeding and adhesion, the cells were treated with the bioavailable and the highest non-toxic concentrations of Abigenol^®^/AlbiPhenol^®^ for 24 h. Afterwards, the treated cells were exposed for 2 h to the oxidation-inducing compound DEM (0.8 mM for HUVEC and H9c2, 5 mM for HepG2). Cells treated with CCM or DEM in the absence of the formulation were considered negative and positive controls, respectively. At the end of the incubation time, the oxidative condition in the in vitro models was assessed by measuring the glutathione state (GSH/GSSG), reactive oxygen species (ROS) and superoxide dismutase (SOD) activity.

### 2.11. Glutathione System (GSH/GSSG) Assay

For the glutathione assay, the GSH/GSSG ratio was determined using a commercially available kit (Promega), following the manufacturer’s instructions. Briefly, both total (GSH + GSSG) and oxidized glutathione (GSSG) measurements are based on the GSH-dependent conversion of a GSH probe, Luciferin-NT, to luciferin by a glutathione S-transferase enzyme. Light from luciferase was dependent on the amount of luciferin formed, which in turn depends on the amount of present GSH. Thus, the luminescent signal is proportional to the GSH amount. Non-oxidized glutathione (GSH) is calculated as the difference between total and oxidized glutathione.

### 2.12. Diclorofluorescein (DCFDA) Assay

To determine the inhibitory effect of Abigenol^®^/AlbiPhenol^®^ on the production of ROS by cells following treatment with DEM, the DCFDA assay was performed. DCFDA (Merck KGaA) is a fluorogenic probe that measures the activity of hydroxyl and peroxyl groups and other ROS within the cell. After intracellular diffusion, DCFDA is deacetylated by cellular esterases to a non-fluorescent compound, which is later oxidized by ROS into 2′,7′-dichlorofluorescein (DCF), a highly fluorescent compound. Briefly, following exposure, the cultured cells were loaded with the DCFDA probe (20 µM) for 1 h. After the removal of the non-internalized probe by washing, fluorescence was detected with a multi-well plate reader (495 nm excitation and 529 nm emission). The obtained results are presented as a percentage compared to the untreated control.

### 2.13. Superoxide Dismutase (SOD) Assay Kit

Following treatment, the cells were detached by trypsin treatment and lysed by sonication in lysis buffer (0.01% Triton X-100 in ddH_2_O). The activity of the SOD enzyme in lysates was determined with a commercial kit, following the manufacturer’s instructions (Merck KGaA). This assay is based on the formation of a colored formazan salt following the reduction of WST-1(2-(4-Iodophenyl)-3-(4-nitrophenyl)-5-(2,4-disulfophenyl)-2H tetrazolium, monosodium salt) by the superoxide anion. The reduction rate with O_2_ is linearly related to the xanthine oxidase (XO) activity and it is inhibited by SOD. The production of the colored formazan salt is monitored by measuring its absorbance at 440 nm. The obtained results were normalized on total protein concentration, as measured by Bradford assay (Bio-Rad).

### 2.14. Determination of Antioxidant Activity on HDL and LDL

The antioxidant activity of the formulation was also evaluated on HDL and LDL by means of a test that monitors over time, by reading the absorbance at 234 nm, the formation of hydroperoxides with conjugated double bonds (conjugated dienes) during the oxidation of fatty acids. HDL or LDL were suspended in PBS at a final concentration of 46.5 and 23.3 μg/mL, respectively. The formulation was added at the bioavailable concentration and at the highest concentration not covering the absorbance signal of the protein. A control with the protein alone, without formulation, was used to monitor their complete oxidation. Subsequently, oxidation was induced by exposure to the pro-oxidant copper sulphate. The reactions were transferred into quartz cuvettes and the kinetics were monitored by reading the absorbance at 234 nm for 12 h, at 5 min intervals and at a temperature of 37 °C. Finally, the curves representing OD values over time were drawn and used to calculate the inhibition of oxidation (%) related to the controls with the proteins alone.

### 2.15. Determination of Antioxidant Activity by ORAC Test

The evaluation of the antioxidant action of the product was further evaluated through the Oxygen Radical Absorbance Capacity (ORAC) test, according to the indications of a commercial kit (Cell Biolabs). The method is based on the incubation of an antioxidant substance with a fluorescent probe, fluorescein, and a free radical initiator that produces peroxyl radicals, resulting in the rapid oxidative degradation of fluorescein and decreased fluorescence. Fluorescence is monitored at one-minute intervals using excitation and emission wavelengths of 480 and 520 nm, respectively. The presence of an antioxidant substance slows down the oxidative degradation of fluorescein, causing a delay in the fluorescence decay. The antioxidant capacity of a substance is related to the fluorescence decay curve and quantified as area under the curve (AUC): a higher antioxidant capacity corresponds to a slower decay of fluorescence and, therefore, to a higher AUC. The AUC is interpolated with a calibration curve obtained with a standard antioxidant substance (Trolox).

The ORAC values are expressed as micromoles of Trolox equivalents per 100 g of product (µMol TE/100 g). The ORAC test was performed following both the protocol for hydrophilic substances, as the extract is soluble in water, and that for lipophilic substances, given the presence of a water-insoluble residue that can be dissolved in acetone. Both hydrophilic and lipophilic fractions were used to determine the antioxidant activity. The total ORAC value is the sum of the hydrophilic and lipophilic fractions’ ORAC values.

### 2.16. Evaluation of Anti-Steatotic Activity of Abigenol^®^/AlbiPhenol^®^ on Hepatocytes

Steatosis was induced in HepG2 cells by treating them with oleic acid. As for the oxidation, cells were treated with the formulation used in a protective approach. The anti-steatotic activity of Abigenol^®^/AlbiPhenol^®^ was evaluated by determining the levels of intracellular lipids, triglycerides, bile acids and cholesterol.

### 2.17. Determination of Intracellular Lipids

The effect of the formulation in reducing intracellular lipid accumulation was evaluated by mimicking a protective approach. HepG2 were treated for 24 h with the formulation, and then for 24 h with the formulation together with two concentrations of oleic acid (0.5 and 1 mM). Intracellular lipid accumulation was quantified using the specific fluorescent dye Nile Red (Merck KGaA, excitation at 525 nm and emission at 600 nm). The intracellular lipid values were then normalized by staining cells with DAPI. The anti-steatotic effect of the formulation was evaluated by comparing the treated and untreated conditions. The Nile Red assay was accompanied by an MTS assay to assess the cell viability under the tested conditions.

### 2.18. Determination of Triglycerides

Triglyceride production was evaluated in the liver model following the protective treatment with the formulation. The used fluorimetric assay (Triglycerides Quantification Kit, Merck KGaA) is based on fluorescent substrate, resorufin production, obtained by the enzymatic conversion of triglycerides into fatty acids and glycerol (excitation at 535 nm and emission at 587 nm). The obtained results were normalized on the concentration of the total proteins in the samples.

### 2.19. Determination of Bile Acids

After exposure to oleic acid and the protective treatment with Abigenol^®^/AlbiPhenol^®^, the cells were lysed by sonication and extracts were employed for bile acid quantification. In the used assay (Bile Acid Assay Kit, Merck KGaA), 3-hydroxysteroid dehydrogenase reacts with the twelve mammalian bile acids, converting NAD to NADH, which reduces a probe to a fluorescent product (excitation at 530 nm and emission at 585 nm). The resulting fluorescence intensity is proportional to the bile acid concentration. The obtained results were normalized on the concentration of the total proteins in the samples.

### 2.20. Determination of Cholesterol

After exposure to oleic acid and the protective treatment with Abigenol^®^/AlbiPhenol^®^, cholesterol was extracted from cells with a micro-homogenization step in a solution made of chloroform, isopropanol and detergent. The Cholesterol Quantitation Kit (Merck KGaA) was used to determine the free cholesterol concentration, cholesteryl esters or both present in samples. Total cholesterol concentration is determined by a coupled enzyme assay, which results in a fluorescent product, proportional to the present cholesterol (excitation at 535 nm and emission at 587 nm). The obtained results were normalized on the concentration of the total proteins in the samples.

### 2.21. Evaluation of Abigenol^®^/AlbiPhenol^®^ Effect on ACE Activity

The impact of Abigenol^®^/AlbiPhenol^®^ on blood pressure regulation at the cardiac level was assessed by measuring its effect on the activity of the enzyme angiotensin-converting enzyme (ACE). Briefly, following H9c2 seeding and adhesion (24 h), the cells were exposed for 24 h to the bioavailable and the highest non-toxic concentrations of Abigenol^®^/AlbiPhenol^®^. H9c2 treated with CCM alone were considered a negative control. After exposure, the cells were washed with PBS and directly lysed with CelLytic M supplemented with EDTA-free protease inhibitors. Cell lysates were centrifuged (15,000 g for 15 min at 4 °C) and the ACE activity of the obtained supernatants determined with a commercial kit (Merck KGaA). The assay is based on the ACE-specific cleavage of a synthetic fluorogenic peptide; measured fluorescence is directly proportional to the ACE activity present. The enzyme activity, expressed in mU, was normalized on the total protein content in lysates, as measured by Bradford assay. Lisinopril was used as a control for ACE inhibition.

### 2.22. Statistical Analysis

The results were analyzed for statistics by using the software OriginLab (OriginLab Corporation, MA, USA). The experiments were performed in triplicate and the results presented as average ± standard deviation. A value of *p* ≤ 0.05 was considered as significant.

## 3. Results

### 3.1. Determination of Abigenol^®^/AlbiPhenol^®^ Concentration

Abigenol^®^/AlbiPhenol^®^ concentration was determined by measuring catechins content, one of the main constituents of this extract. In particular, the measured amount of catechins on the used batch was 9.2 ± 0.1 mg/dose.

### 3.2. Abigenol^®^/AlbiPhenol^®^ Intestinal Absorption

**Figure 1:**
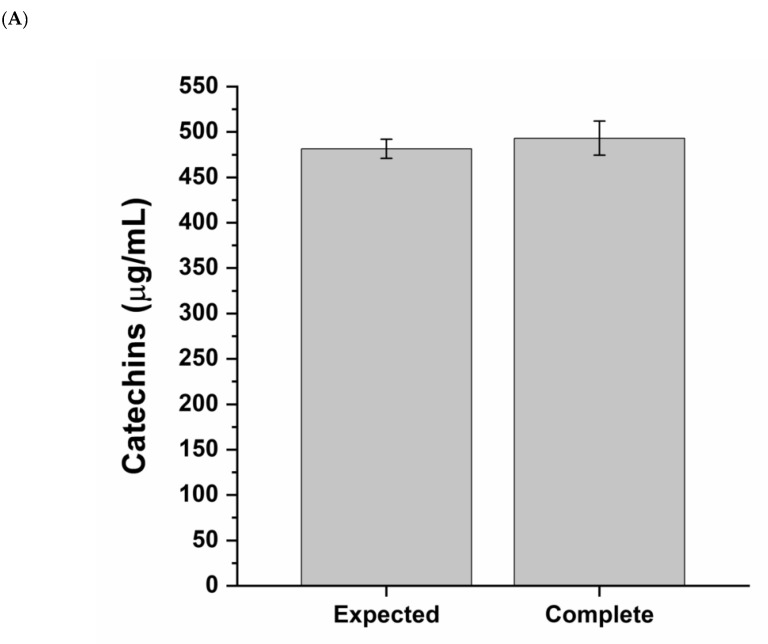

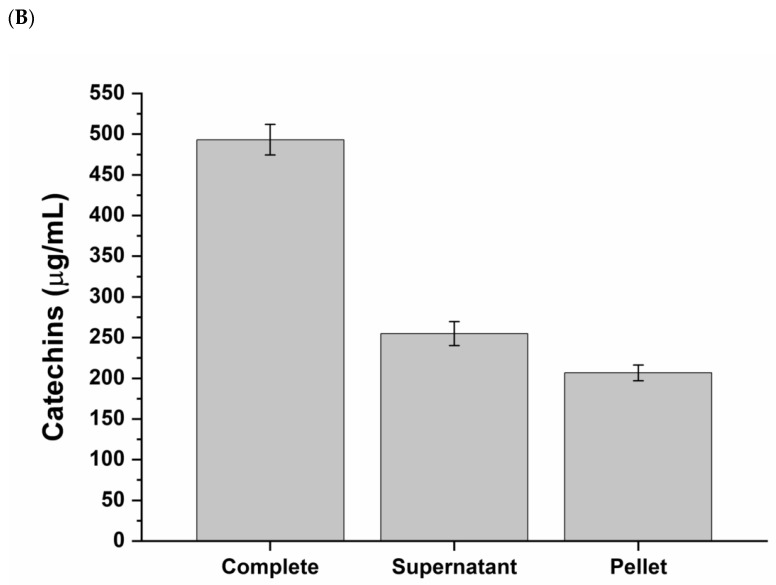
Catechins content of Abigenol^®^/AlbiPhenol^®^ (**A**) at the end of the processing phase and (**B**) in the supernatant and pellet fractions.

The bioaccessibility of a dietary supplement refers to the active components released from its matrix in a form available for absorption. To determine the bioaccessible fraction of Abigenol^®^/AlbiPhenol^®^, a single dose (150 mg) was exposed to an in vitro digestion procedure mimicking the human digestive process in the adult. Then, the total amount of catechins was compared to the amount of soluble catechins released from the matrix (bioaccessible fraction, which includes the portion available for absorption). As can be seen in Figure 1 and Table 1, Abigenol^®^/AlbiPhenol^®^ is endowed with good bioaccessibility, considering the lipophilic nature of its active compound, since about the 50% (255.0 ± 14.5 µg/mL) of catechins contained in the extract was released from the matrix during the digestion (Figure 1B). Furthermore, Abigenol^®^/AlbiPhenol^®^ was highly resistant to the digestive process. Indeed, the amount of catechins measured at the end of the in vitro digestive process was equivalent to the expected amount (493.2 ± 18.7 vs. 481.6 ± 10.7 µg/mL) (Figure 1A). However, only a relevant catechin fraction (about 43%) was non-bioaccessible and excreted through the feces (Figure 1B). 

**Figure 2:**
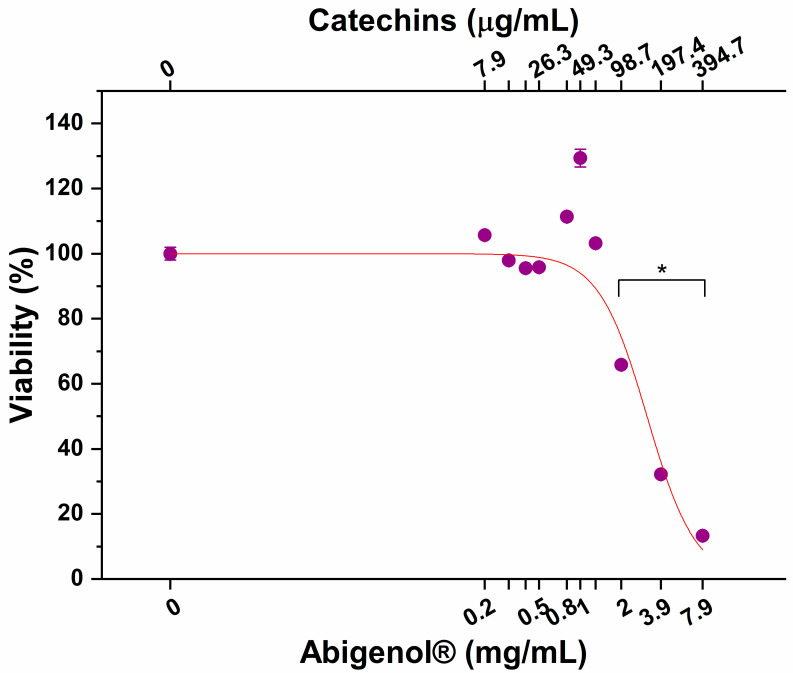
Impact of increasing concentrations of digested Abigenol^®^/AlbiPhenol^®^ on the vitality of the intestinal epithelium. * *p* < 0.05. (n = 3).

**Figure 3:**
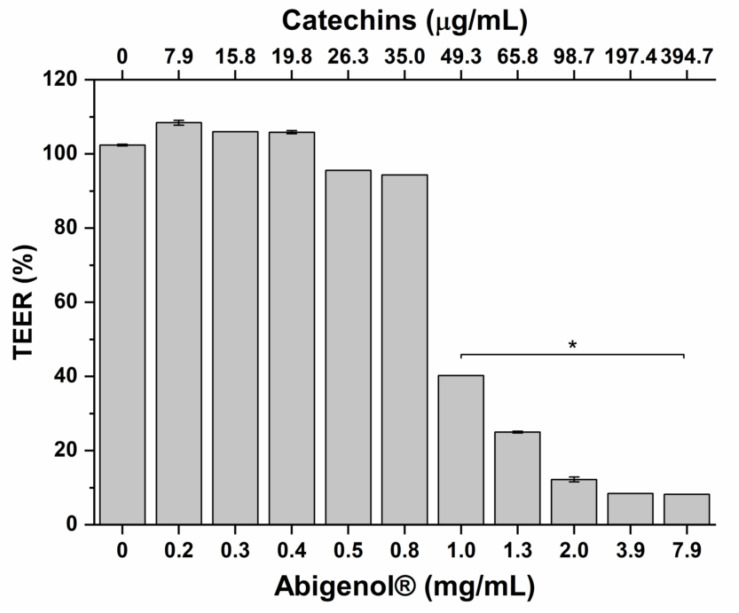
Impact of increasing concentrations of digested Abigenol^®^/AlbiPhenol^®^ on the barrier integrity of the intestinal epithelium. * *p* < 0.05. (n = 3).

The bioaccessible fraction of Abigenol^®^/AlbiPhenol^®^ released from the matrix was tested for intestinal absorption using the well-characterized in vitro Caco-2 model. Prior to performing this assay, the impact of bioaccessible fraction on intestinal mucosa viability was evaluated. This aspect has to be taken into consideration because damages to the intestinal epithelium may lead to a decrease in absorption efficiency. To this aim, intestinal monolayers were exposed to increasing concentrations of Abigenol^®^/AlbiPhenol^®^ and a dose–response curve was obtained. As shown in Figure 2, no effect on the intestinal epithelium vitality was observed up to a concentration of 1.3 mg/mL of Abigenol^®^/AlbiPhenol^®^, equivalent to 65.8 µg/mL of catechins, expressed as EGCG. The same Abigenol^®^/AlbiPhenol^®^ concentrations significantly affected the barrier integrity of the intestinal epithelium model, as highlighted in Figure 3. No effect on the intestinal epithelium barrier integrity was observed up to 0.8 mg/mL of Abigenol^®^/AlbiPhenol^®^, equivalent to 35.0 µg/mL of catechins. To avoid any overestimation of Abigenol^®^/AlbiPhenol^®^ bioavailability due to alterations of the barrier integrity, the epithelia were exposed to digested Abigenol^®^/AlbiPhenol^®^ at a concentration of 0.8 mg/mL, equivalent to 35.0 µg/mL of catechins. After having determined the impact of digested Abigenol^®^/AlbiPhenol^®^ on intestinal epithelium viability and integrity, the monolayers were exposed to digested Abigenol^®^/AlbiPhenol^®^ for 1 and 3 h and, thereafter, catechins were measured in both apical (lumen) and basolateral (serosal) chambers. Then, the bioavailability was calculated and expressed as a percentage of absorption. Despite the good Abigenol^®^/AlbiPhenol^®^ bioaccessibility, the bioavailable fraction of catechins in the basolateral compartment was below the HPLC detection limit (LOD = 2.7 µg/mL). Consequently, Abigenol^®^/AlbiPhenol^®^ intestinal absorption seems to be limited, at least considering the experimental setup described here.

### 3.3. Impact of Digested Formulations on Intestinal Mucosa Viability and Integrity

**Figure 4:**
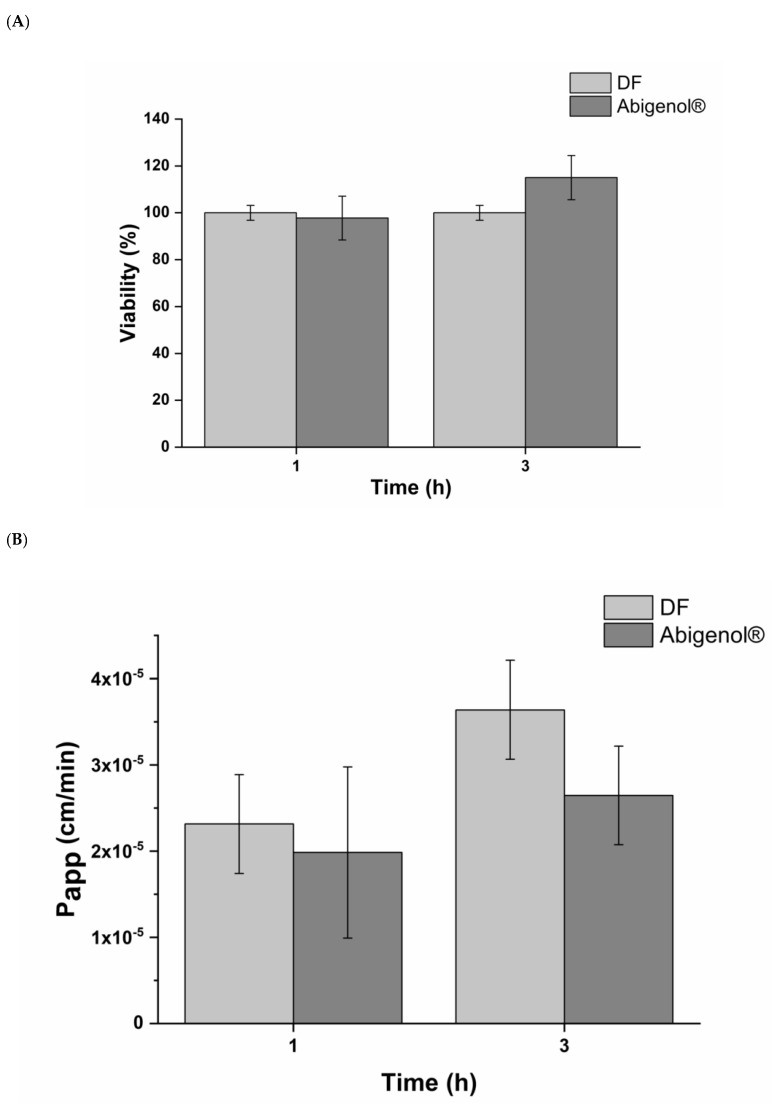
Cell vitality (**A**) and apparent permeability (Papp) (**B**) of intestinal epithelia exposed to digestive fluids (DF; control) and digested Abigenol^®^/AlbiPhenol^®^ (n = 3).

**Figure 5:**
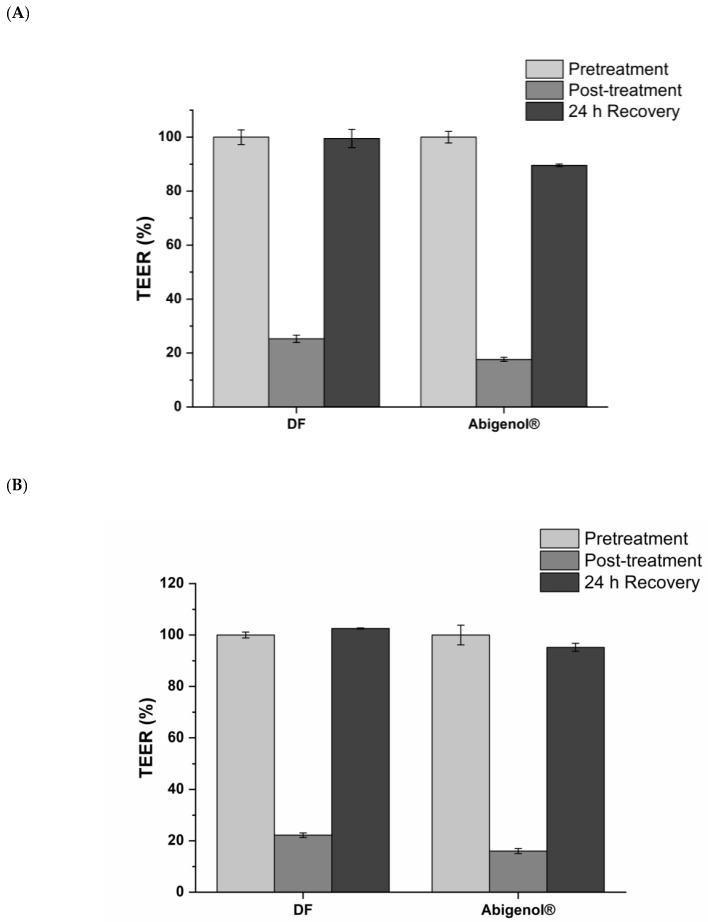
TEER values before the treatment (pre-treatment), after exposure to digestive fluids (DF) or digested Abigenol^®^/AlbiPhenol^®^ (post-treatment) for 1 h (**A**) and 3 h (**B**) and after 24 h of recovery. Values are expressed as a percentage of the pre-treatment TEER value (n = 3).

After exposure of the intestinal epithelia to digested Abigenol^®^/AlbiPhenol^®^, the viability and barrier integrity of the intestinal monolayer were analyzed. As expected, no viability reduction (Figure 4A) or apparent permeability (Papp) (Figure 4B) were observed during the bioavailability experiments. A prolonged alteration of the intestinal barrier integrity was also ruled out by TEER measurement. Indeed, as shown in Figure 5A,B, the TEER alteration provoked by treatment with digestive fluids or digested Abigenol^®^/AlbiPhenol^®^ was only transitory, since the pre-treatment values were fully recovered after 24 h. Considering the obtained result, Abigenol^®^/AlbiPhenol^®^ appeared to be a safe formulation that did not affect either the vitality or barrier integrity of the intestinal epithelium.

### 3.4. Evaluation of Abigenol^®^/AlbiPhenol^®^ Cytotoxicity

**Figure 6:**
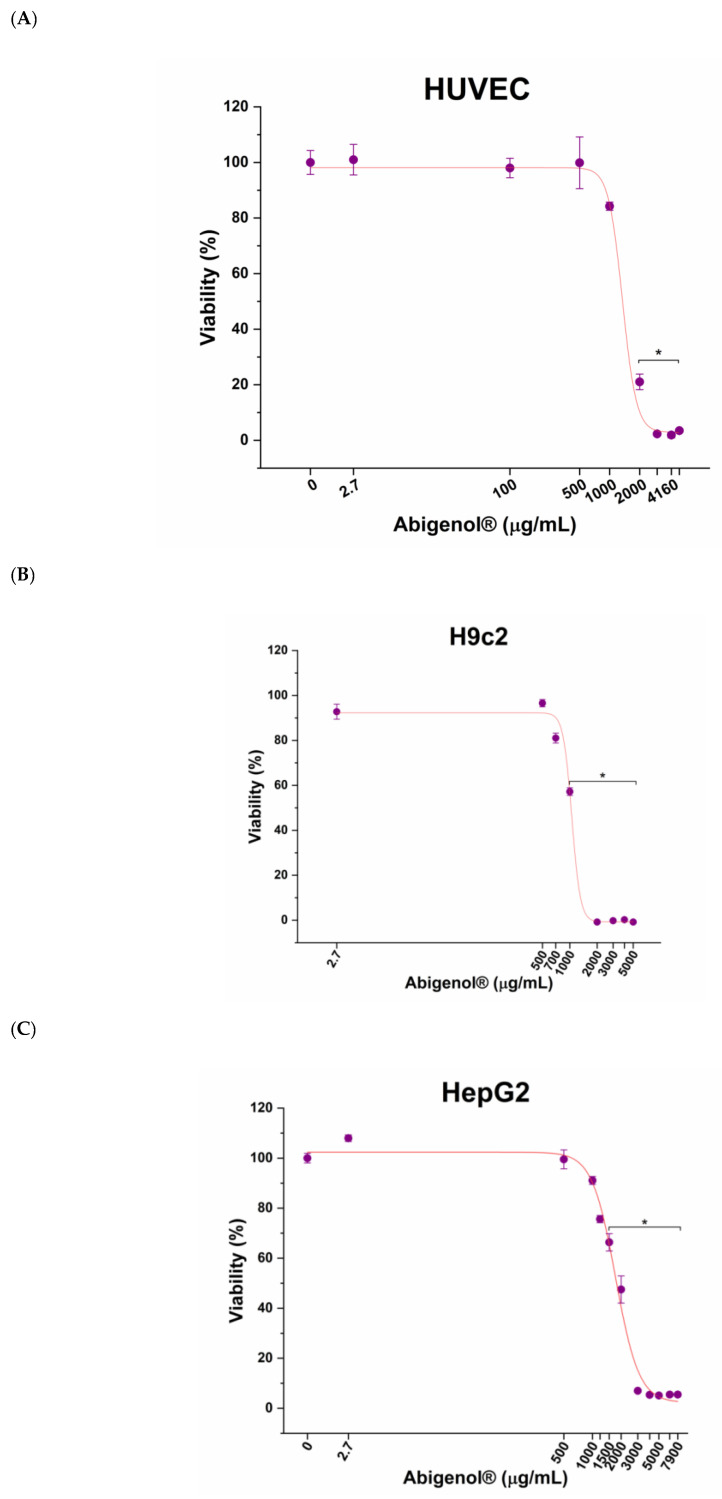
Impact of increasing concentrations of Abigenol^®^/AlbiPhenol^®^ on in vitro HUVEC cells (**A**), H9c2 cells (**B**) and HepG2 cells (**C**). * *p* < 0.05 (n = 3).

In order to verify that Abigenol^®^/AlbiPhenol^®^ could not negatively affect target organs and tissues, a cytotoxicity analysis was performed before efficacy tests on cultured cells. To this aim, the cells were exposed to increasing concentrations of Abigenol^®^/AlbiPhenol^®^ (including the bioavailable one, 2.7 μg/mL) and dose–response curves were obtained (Figure 6). In the case of HUVEC cells, the maximum non-toxic concentration of Abigenol^®^/AlbiPhenol^®^ resulted in 1000 µg/mL; higher concentrations significantly lowered the vitality below 70% (Figure 6A). Similarly, the maximum non-toxic concentrations for H9c2 and HepG2 cells were 700 µg/mL and 1200 μg/mL, respectively (Figure 6B,C). For all three cell lines, the bioavailable concentration of Abigenol^®^/AlbiPhenol^®^ resulted in being non-toxic (101.0 ± 5.5% for HUVEC, 108.0 ± 1.3% for HepG2 and 92.8 ± 3.3% for H9c2, compared to the untreated control). To better explore the effects of Abigenol^®^/AlbiPhenol^®^ on the three in vitro models, both the bioavailable concentration and the maximum non-toxic concentrations were used. 

### 3.5. Determination of Antioxidant Activity of Abigenol^®^/AlbiPhenol^®^

**Figure 7:**
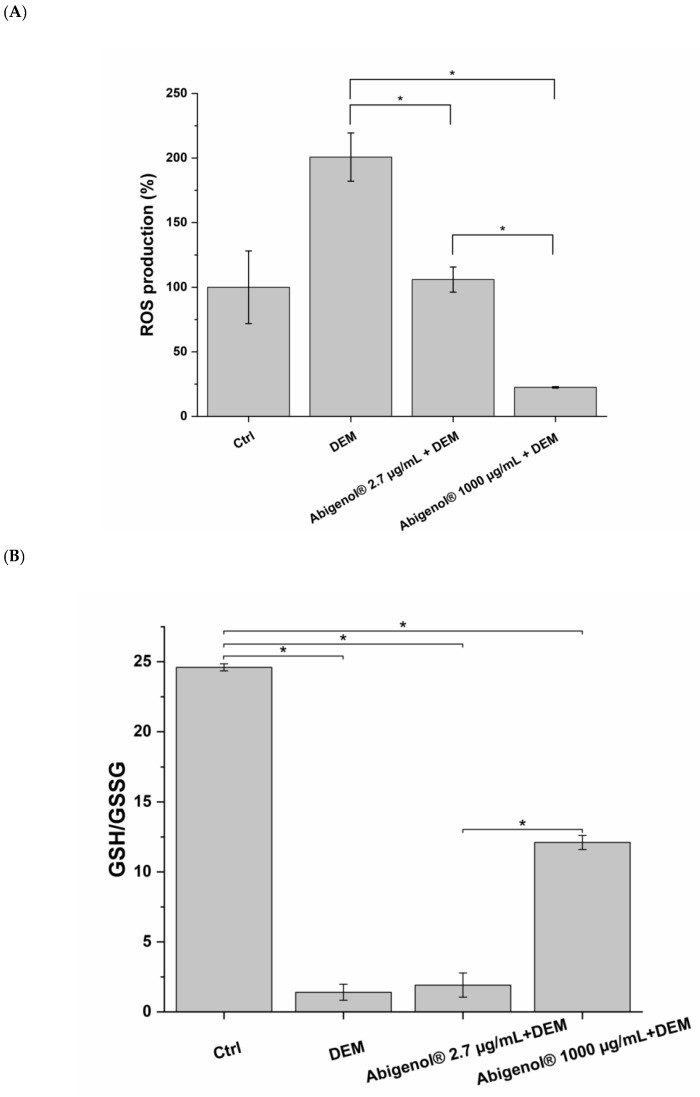

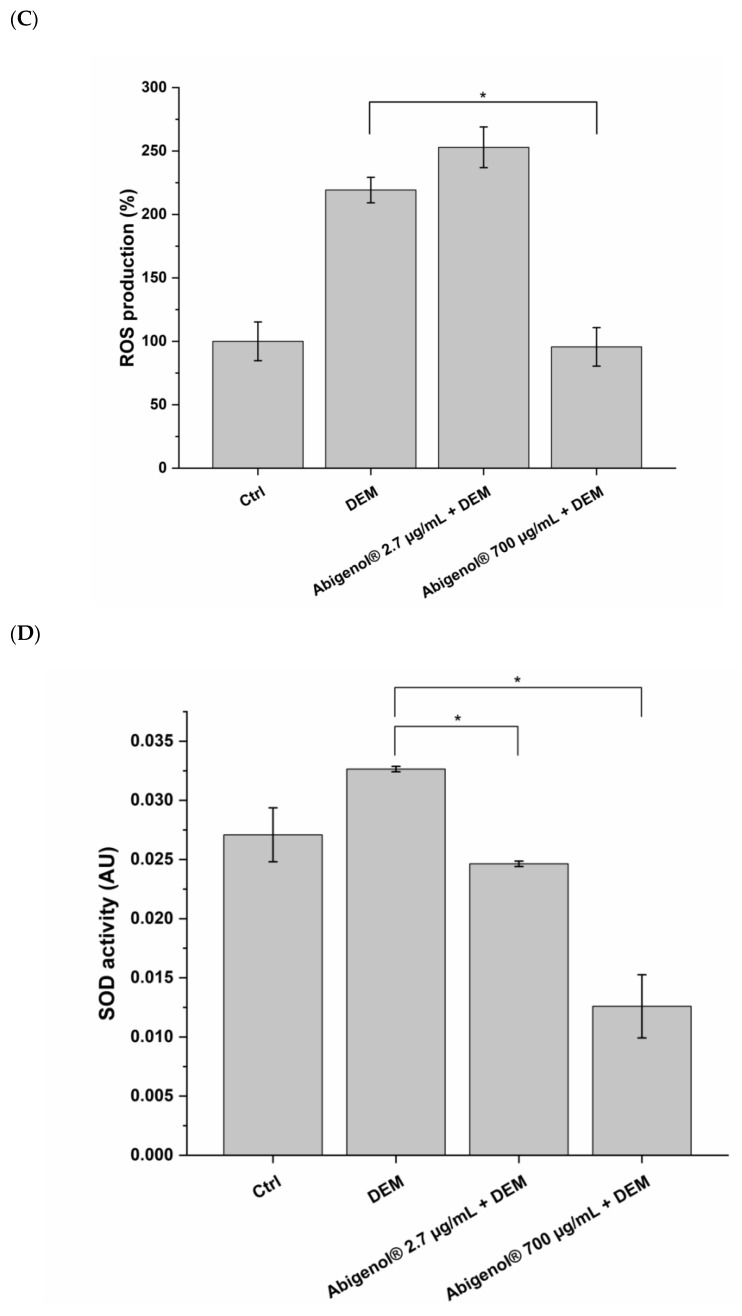

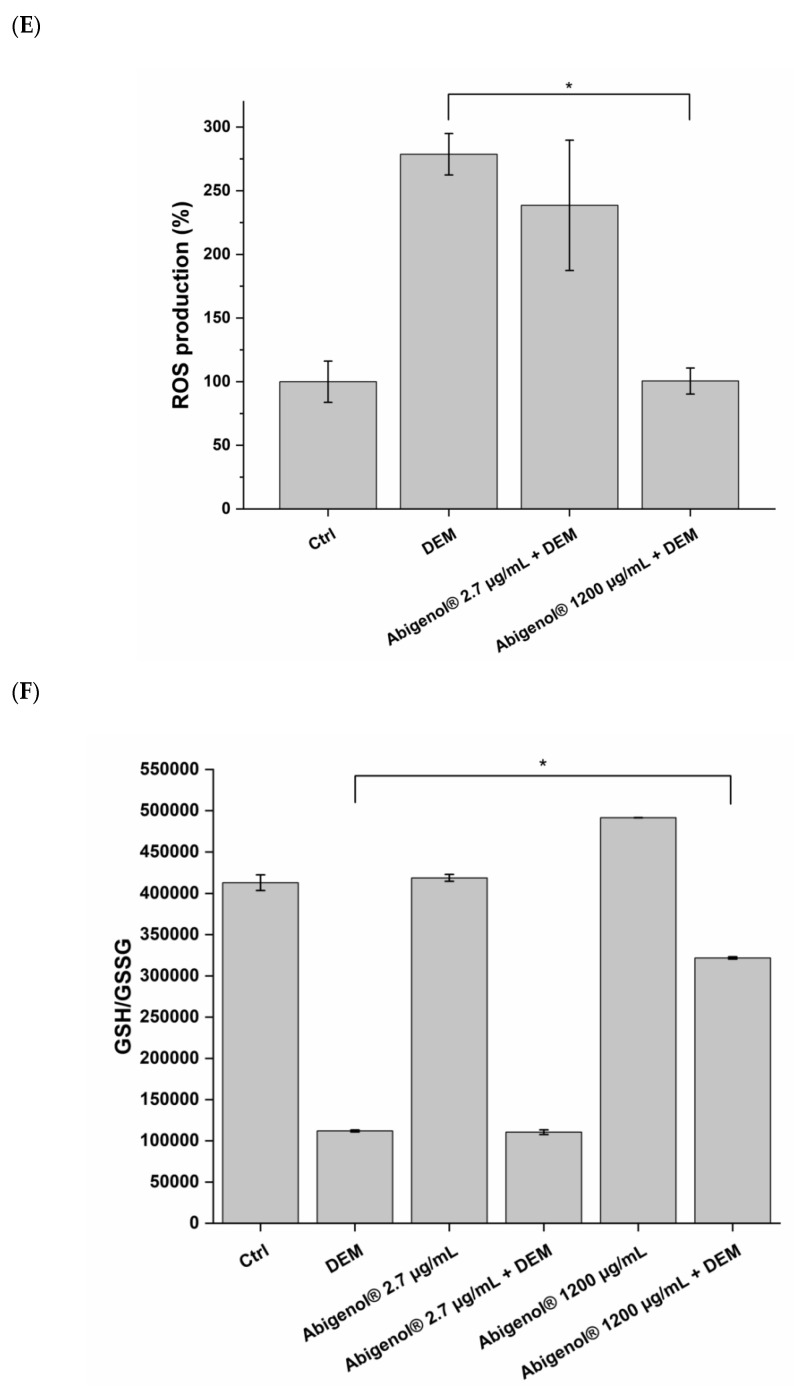
Impact of Abigenol^®^ on the cellular oxidative state, assessed as production of ROS, glutathione oxidation (GSH/GSSG ratio) or SOD activity of HUVEC (**A**,**B**), H9c2 (**C**,**D**) and HepG2 (**E**,**F**). Cells were incubated for 24 h in the presence of the formulation, followed by 2 h with the formulation + DEM. ROS were normalized on DAPI and expressed as fold-change of the untreated control; glutathione was normalized on total proteins in samples. Ctrl: control (untreated cells); DEM: diethylmaleate; AU: arbitrary units. * *p* < 0.05. (n = 3).

**Figure 8:**
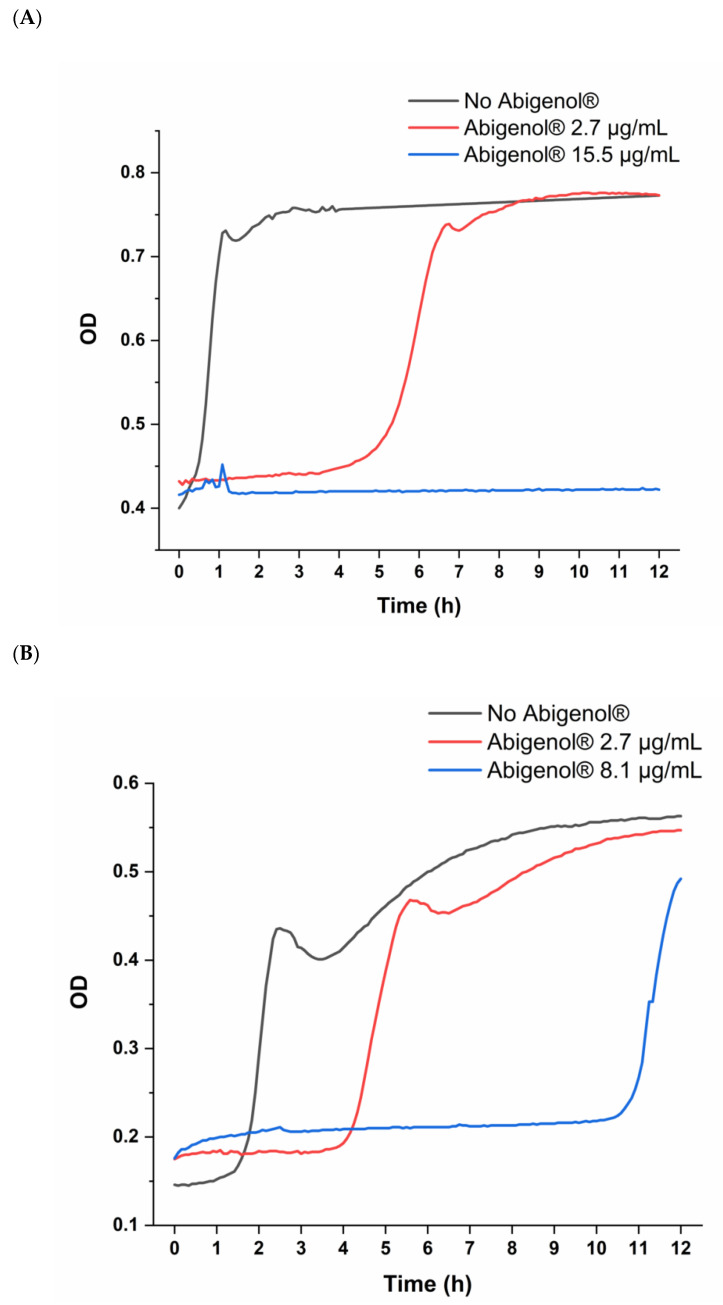
Effects of the silver fir extract on the oxidation of HDL (**A**) and LDL (**B**), induced by exposure to the pro-oxidant copper sulphate. The kinetics were monitored by reading the absorbance at 234 nm for 12 h at 5 min intervals.

The overall cardiovascular function depends on the balance between oxidant and antioxidant mechanisms. Indeed, ROS production and oxidative stress are associated with several pathological conditions (e.g., hypercholesterolemia and diabetes) and have a pivotal role in the onset of CVDs, in particular CADs and HF. As already mentioned above, oxidative damages to the vascular endothelium, oxidation of circulating LDL/HDL and steatosis-induced production of ROS in the liver may cause atherosclerosis and, consequently, CADs. In addition, oxidative stress has also been linked to hypertension and, therefore, to HF [37,38]. The antioxidant activity of the extract was assessed on cultured cells, as well as on the lipoproteins HDL and LDL. Moreover, this activity was quantified through the ORAC test. To investigate possible Abigenol^®^/AlbiPhenol^®^ antioxidant effects on the in vitro models, the cells were pretreated with both the bioavailable and the maximum non-toxic concentrations of Abigenol^®^/AlbiPhenol^®^, and then exposed to the pro-oxidant agent diethylmaleate (DEM). The antioxidant effect of the formulation was evaluated in terms of ROS production, glutathione oxidation state and SOD activity. In the case of HUVEC cells (Figure 7A,B), Abigenol^®^/AlbiPhenol^®^ effectively reduced the oxidative load produced by DEM in the endothelium. Indeed, the fir bark extract decreased both ROS production and glutathione oxidation. However, while the Abigenol^®^/AlbiPhenol^®^ bioavailable concentration (2.7 µg/mL) was sufficient to significantly reduce DEM-induced ROS production, a significant increase in the GSH/GSSG ratio (i.e., a reduction in glutathione oxidation) was only observed at the highest non-toxic concentration of Abigenol^®^/AlbiPhenol^®^ (1000 µg/mL). No significant changes in superoxide dismutase (SOD) expression were highlighted following endothelium exposure to Abigenol^®^/AlbiPhenol^®^ (data not shown). Abigenol^®^/AlbiPhenol^®^ also reduced the oxidative stress produced by DEM in H9c2 cells, since the extract decreased ROS production (Figure 7C) and SOD activity (Figure 7D). In particular, the maximum non-toxic Abigenol^®^/AlbiPhenol^®^ concentration (700 µg/mL) significantly reduced both SOD activity and ROS production, while the bioavailable concentration only reduced SOD activity. No effects on the glutathione system were observed at either of the tested Abigenol^®^/AlbiPhenol^®^ concentrations (data not shown). Finally, Abigenol^®^/AlbiPhenol^®^ at 1200 µg/mL effectively reduced oxidation in HepG2 cells, since the extract decreased ROS production (Figure 7E) and increased the GSH/GSSG ratio (Figure 7F) when compared to DEM alone. The antioxidant activity of the formulation was also evaluated on the lipoproteins HDL and LDL by means of a kinetic test that monitors, over time, the formation of conjugated dienes in oxidative conditions. Abigenol^®^/AlbiPhenol^®^ was used at the bioavailable concentration and, for each tested lipoprotein, at the highest concentration that was compatible with the assay (15.5 µg/mL for HDL and 8.1 µg/mL for LDL). As shown in Figure 8 and Table 2, the bioavailable Abigenol^®^/AlbiPhenol^®^ concentration was able to significantly delay the oxidation of both HDL and LDL (51.8% and 43.9% of inhibition on HDL and LDL, respectively). This antioxidant activity was further evidenced at higher Abigenol^®^/AlbiPhenol^®^ concentrations, as shown in Figure 8 and Table 2, with an inhibition of oxidation equal to 98.5% and 85.8% for HDL and LDL, respectively. 

The antioxidant properties of Abigenol^®^/AlbiPhenol^®^ were also evaluated through the ORAC assay, in which the mechanism is based on quenching peroxyl free radicals, the major oxidative products found during lipid peroxidation in biological systems [39]. The results, expressed as micromoles of Trolox equivalents per 100 g of product (µMol TE/100 g), are reported in Table 3. Total-ORAC represents the sum of the hydrophilic and the lipophilic fractions values of the formulation (H-ORAC and L-ORAC, respectively). As reported in Table 4, the main contribution derived from the hydrophilic fraction.

In order to understand the antioxidant activity of Abigenol^®^/AlbiPhenol^®^, it is possible to compare the obtained total-ORAC value with the database for the ORAC of selected foods published by the USDA (US Department of Agriculture) [40]. Abigenol^®^/AlbiPhenol^®^ places itself among the first four foods with the highest ORAC values in the database, which currently lists 326 foods (Table 4).

### 3.6. Evaluation of Anti-Steatotic Activity of Abigenol^®^/AlbiPhenol^®^

**Figure 9:**
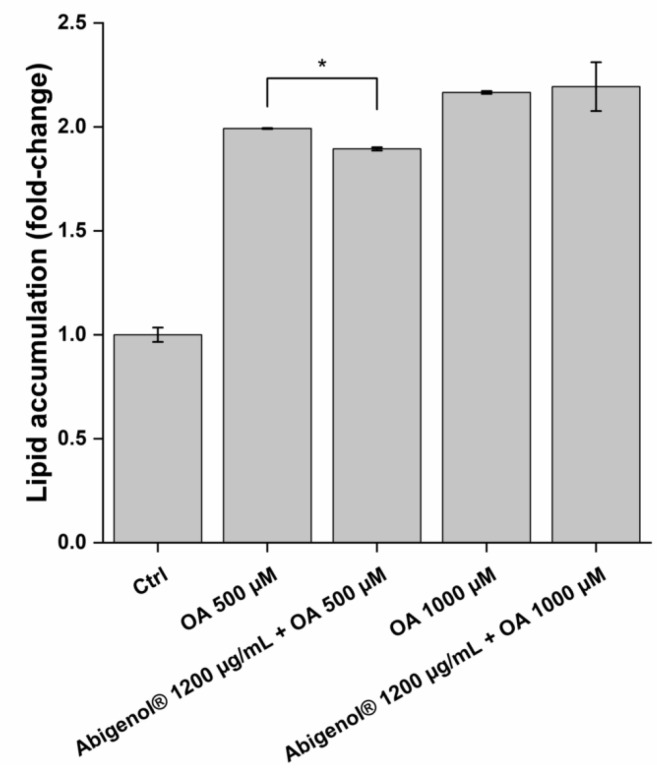
Intracellular lipid accumulation in HepG2 cells after a protective treatment with Abigenol^®^/AlbiPhenol^®^ (24 h with the formulation, followed by 24 h with the formulation + oleic acid). Intracellular lipids were normalized on DAPI. AU: arbitrary units; Ctrl: control (untreated cells); OA: oleic acid. * *p* < 0.05. (n = 3).

**Figure 10:**
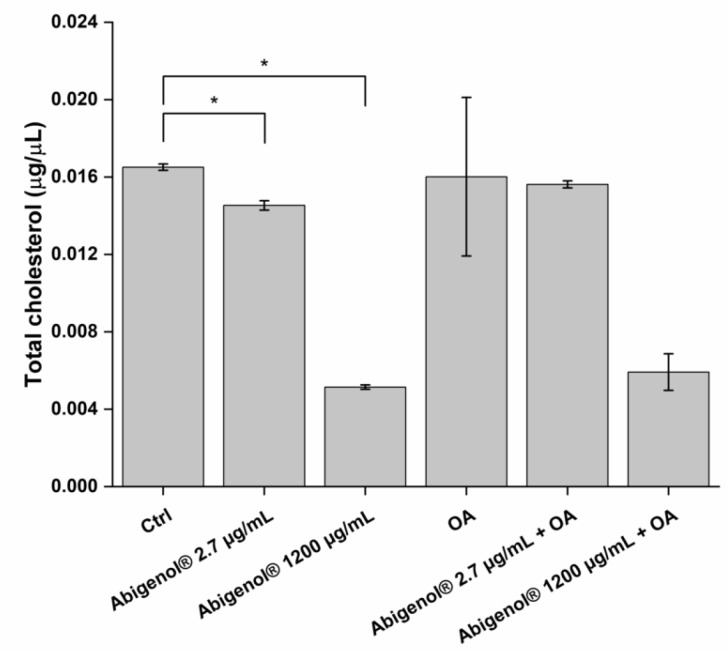
HepG2 total cholesterol in physiological and steatotic conditions after a protective treatment with Abigenol^®^/AlbiPhenol^®^ (24 h with the formulation, followed by 24 h with the formulation + oleic acid). Cholesterol was normalized on the amount of total proteins in samples. Ctrl: control (untreated cells); OA: oleic acid. * *p* < 0.05.

Hepatic steatosis is one of the major causes of liver dysfunction and disease. To investigate the extract’s impact on this process, the HepG2 liver cell line was treated with Abigenol^®^/AlbiPhenol^®^ in a protective approach, as for the oxidative stress; then, the cells were exposed to oleic acid, a well-known inducer of fat and lipid accumulation. The anti-steatotic activity of Abigenol^®^/AlbiPhenol^®^ was evaluated by determining the HepG2 intracellular levels of lipids, cholesterol, bile acids and triglycerides. Two different concentrations of oleic acid were used (0.5 and 1 mM) and the highest non-toxic concentration of the extract was tested (1200 µg/mL). As shown in Figure 9, Abigenol^®^/AlbiPhenol^®^ was able to significantly reduce the hepatic lipid accumulation induced by oleic acid used at 0.5 mM, but not at 1 mM. Afterwards, cholesterol was extracted from the hepatocytes and quantified (Figure 10 and Table 5). In physiological conditions (the absence of oleic acid-induced steatosis), the extract at both tested concentrations significantly reduced the total cholesterol concentration compared to the untreated control. A concentration-dependent effect was observed, since there was a decrease of cholesterol equal to 12.0% and 68.8% following treatment with 2.7 and 1200 µg/mL of Abigenol^®^/AlbiPhenol^®^, respectively (Figure 10 and Table 5). However, when HepG2 cells were exposed to 0.5 mM oleic acid (steatotic conditions), the total cholesterol was only significantly lowered with Abigenol^®^/AlbiPhenol^®^ at 1200 µg/mL, while no effect was observed with the bioavailable concentration (Figure 10 and Table 5). Most of the measured cholesterol was represented by free cholesterol, while esterified cholesterol was a very limited fraction. The ratio between free and esterified cholesterol was not affected by Abigenol^®^/AlbiPhenol^®^ and/or oleic acid treatment (data not shown).

**Figure 11:**
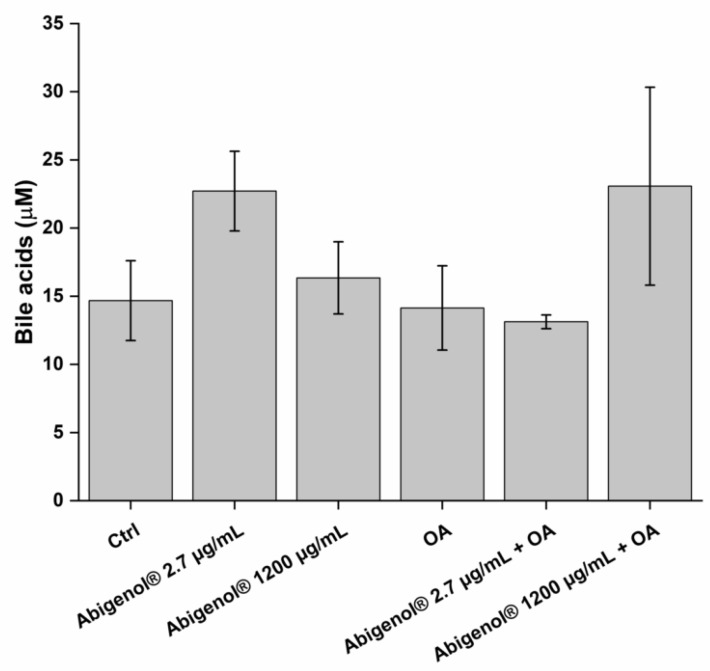
Bile acids in HepG2 cells after a protective treatment with Abigenol^®^/AlbiPhenol^®^ (24 h with the formulation, followed by 24 h with the formulation + oleic acid). Bile acids were normalized on protein content in samples. Ctrl: control (untreated cells); AO: oleic acid. (n = 3).

Cholesterol conversion to bile acids plays a key role in hepatic cholesterol homeostasis, and is key in the elimination of cholesterol, which is one of the main factors regulating cholesterol homeostasis in the body. In order to evaluate whether Abigenol^®^/AlbiPhenol^®^ could promote the bioconversion of cholesterol, bile acids were quantified inside the cells. In physiological conditions, Abigenol^®^/AlbiPhenol^®^ induced an increase in the bile acid level at the bioavailable concentration, while in steatotic conditions, it was necessary to use the higher Abigenol^®^/AlbiPhenol^®^ concentration (1200 µg/mL) to obtain a similar variation (Figure 11 and Table 6).

### 3.7. Evaluation of Abigenol^®^/AlbiPhenol^®^ Effect on ACE Activity

**Figure 12:**
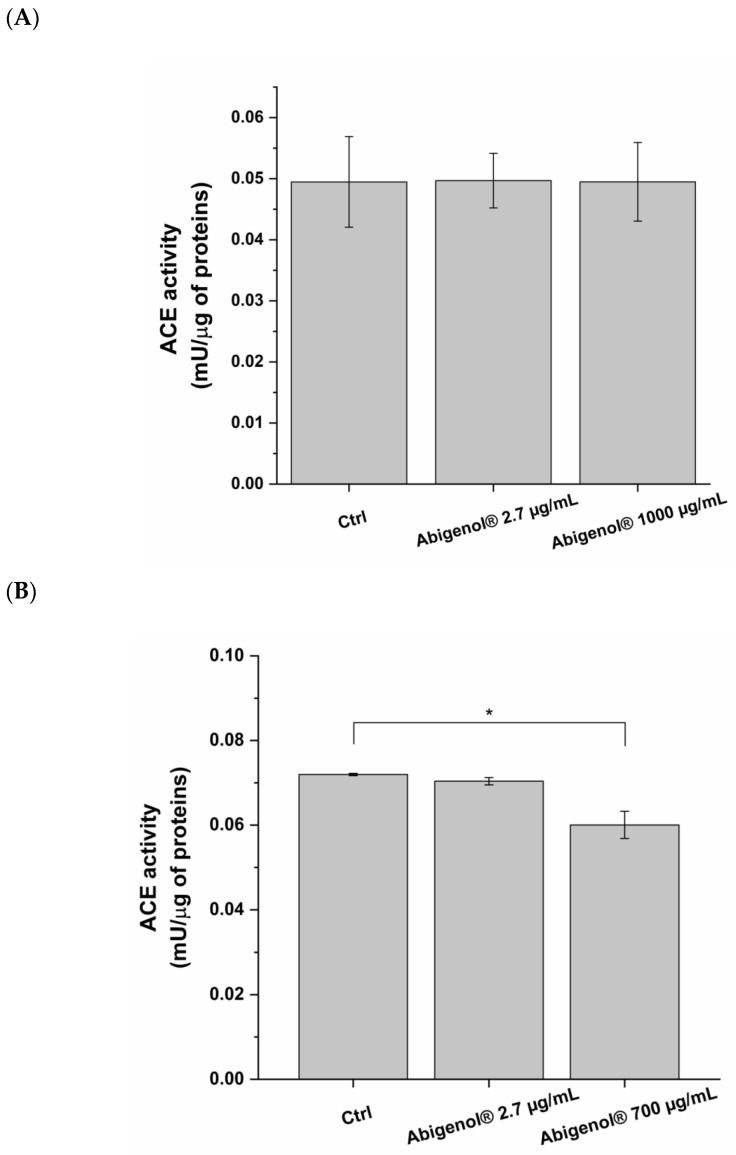
Effect of the silver fir extract on ACE activity in HUVEC (**A**) and H9c2 (**B**). Data were normalized on total proteins present in samples. Lisinopril was used as a control for ACE inhibition. Ctrl: control (untreated cells). * *p* < 0.05. (n = 3).

One of the major risk factors for CVDs is arterial hypertension, which accelerates the progression of atherosclerosis and cardiovascular events such as HF. The activation of the RAAS system plays a key role in arterial hypertension pathogenesis. Indeed, the ACE-mediated conversion of the hormone angiotensin I into angiotensin II promotes blood vessel constriction and blood pressure increase. Since ACE is expressed in both endothelial cells and cardiomyocytes [41,42], the potential ability of Abigenol^®^/AlbiPhenol^®^ to inhibit ACE activity in vitro was investigated in order to understand whether it might be able to reduce vasoconstriction in vivo. As shown in Figure 12, the maximum non-toxic concentration was able to significantly reduce ACE activity in H9c2, but not in HUVEC. The bioavailable concentration had no significant effects on either cell line.

## 4. Discussion

In the last decades, the potential therapeutic effects of silver fir extracts have catalyzed the interest of the nutraceutical and food supplement industries. Indeed, silver fir extracts are beneficial to human health, since they exert anti-inflammatory and antioxidant effects. These effects may be relevant for the prevention and treatment of CVDs, such as atherosclerosis and HF. Indeed, it has been demonstrated that atherosclerotic plaque formation is linked to inflammatory and oxidative stress. The positive impact of these extracts on human health may be reduced when they are ingested, due to the poor solubility of the main constituents (polyphenols and catechins) in aqueous fluids, such as those of the digestive process. Consequently, their bioaccessibility and bioavailability could also be limited. Here, Abigenol^®^/AlbiPhenol^®^ showed good bioaccessibility, indicating a good release of the active compounds from the matrix during the digestive process. However, considering our experimental setup, Abigenol^®^/AlbiPhenol^®^ seems to have limited bioavailability, indicating low intestinal absorption. Abigenol^®^/AlbiPhenol^®^ was shown to be safe, since no effect on intestinal epithelium vitality and barrier integrity was observed. Then, the effects of Abigenol^®^/AlbiPhenol^®^ to inhibit oxidative stress were investigated at the endothelial, hepatic and cardiac levels. Abigenol^®^/AlbiPhenol^®^ effectively reduced the oxidative load produced by DEM in the three in vitro models. Indeed, the fir bark extract decreased ROS production and glutathione oxidation in endothelial cells and hepatocytes, while it reduced ROS production and SOD activity in cardiac myoblasts. Considering the three models, the observed antioxidant effects were more intense at the highest non-toxic concentration than at the bioavailable concentration. Abigenol^®^/AlbiPhenol^®^ also reduced HDL and LDL oxidation, thus suggesting it is instrumental in limiting atherosclerotic plaque formation in vivo. As indicated by the ORAC test, all of these antioxidant effects are probably due to Abigenol^®^/AlbiPhenol^®^ composition, enriched in polyphenols and catechins. Moreover, according to the link between cardiovascular diseases and liver steatosis, the anti-steatotic properties of Abigenol^®^/AlbiPhenol^®^ were investigated. The extract was found to be endowed with an interesting protective effect at the highest non-toxic concentration, since it reduced intracellular lipids and total cholesterol accumulation in steatotic conditions. Furthermore, Abigenol^®^/AlbiPhenol^®^ also stimulated a slight production of bile acids, showing the potential ability to stimulate the degradation of cholesterol in the liver. Another positive property of Abigenol^®^/AlbiPhenol^®^ was highlighted in cardiomyocytes cell model H9c2, where the maximum non-toxic concentration was able to significantly reduce ACE activity because in vivo this could be associated with a decrease in hypertension risk. Finally, Abigenol^®^/AlbiPhenol^®^ was found to be safe when used at the bioavailable concentration, since no effects on the vitality of in vitro endothelial cells, hepatocytes and myoblasts were observed. 

## 5. Conclusions

In conclusion, Abigenol^®^/AlbiPhenol^®^ has proven to exhibit effective antioxidant and antisteatotic activity in tested conditions, and it significantly reduces ACE activity in cultured cardiac cells. Further in vivo and in vitro studies should be undertaken in order to provide a better understanding of the potential contribution of Abigenol^®^/AlbiPhenol^®^ in preventing the spectrum of cardiovascular diseases.

## Figures and Tables

**Table 1:** Abigenol^®^/AlbiPhenol^®^ catechin (CTCN) content at the end of the processing phase and in the supernatant (bioaccessible fraction) and pellet (excreted fraction). Percentages are calculated on the expected catechin concentration at the end of the digestive process. Results are shown as average ± standard deviation of three replicates (n = 3).

	Complete		Supernatant		Pellet	
	(CTCN) (µg/mL)	CTCN (%)	(CTCN) (µg/mL)	CTCN (%)	(CTCN) (µg/mL)	CTCN (%)
**Abigenol^®^/AlbiPhenol^®^**	493.4 ± 18.7	102.4 ± 3.9	255.0 ± 14.5	53.0 ± 3.0	206.9 ± 9.7	43.0 ± 2.0

**Table 2:** Inhibition of oxidation (%) for HDL and LDL in the presence of the extract compared to the controls (proteins without the extract).

	Inhibition of Oxidation (%)	
Concentration of Abigenol^®^/AlbiPhenol^®^	HDL	LDL
2.7 µg/mL	51.8	43.9
15.5 µg/mL (HDL) or 8.1 µg/mL (LDL)	98.5	85.8

**Table 3:** Mean ORAC values calculated as micromoles of Trolox equivalents per 100 g of formulation (µmol TE/100 g) on both the hydrophilic (H-ORAC) and the lipophilic (L-ORAC) fractions of the formulation. Total-ORAC represents the sum of H-ORAC and L-ORAC.

Amount of Abigenol^®^/AlbiPhenol^®^/mL	Mean ORAC Value (µMol TE/100 g)
H-ORAC	221,359
L-ORAC	10,538
Total-ORAC	231,897

**Table 4:** Total-ORAC value of Abigenol^®^/AlbiPhenol^®^ compared to those of the most antioxidant foods according to the database for the ORAC of selected foods (*) (US Department of Agriculture) (39).

Food	Mean ORAC Value (µMol TE/100 g)
Sumac, bran, raw	312,400 *
Spices, cloves, ground	290,283 *
Sorghum, bran, hi-tannin	240,000 *
Abigenol^®^/AlbiPhenol^®^	231,897

**Table 5:** Total cholesterol decrease (%) in the presence of the extract compared to the untreated controls.

	Total Cholesterol Decrease (%)	
Concentration of Abigenol^®^/AlbiPhenol^®^	Physiological Conditions	Steatosis-Inducing Conditions
2.7 µg/mL	12.0	2.5
1200 µg/mL	68.8	63.0

**Table 6:** Bile acid increase (%) in the presence of the extract compared to the untreated controls.

	Bile Acid Increase (%)	
Concentration of Abigenol^®^/AlbiPhenol^®^	Normal Conditions	Steatosis
2.7 µg/mL	54.7	−7.2
1200 µg/mL	11.4	63.2

## Data Availability

All of the data is contained within the article.
